# An accurate and precise representation of drug ingredients

**DOI:** 10.1186/s13326-016-0048-2

**Published:** 2016-04-19

**Authors:** Josh Hanna, Jiang Bian, William R. Hogan

**Affiliations:** Biomedical Informatics Program, Department of Health Outcomes and Policy, University of Florida, Gainesville, FL USA

## Abstract

**Background:**

In previous work, we built the Drug Ontology (DrOn) to support comparative effectiveness research use cases. Here, we have updated our representation of ingredients to include both active ingredients (and their strengths) and excipients. Our update had three primary lines of work: 1) analysing and extracting excipients, 2) analysing and extracting strength information for active ingredients, and 3) representing the binding of active ingredients to cytochrome P450 isoenzymes as substrates and inhibitors of those enzymes.

**Methods:**

To properly differentiate between excipients and active ingredients, we conducted an ontological analysis of the roles that various ingredients, including excipients, have in drug products. We used the value specification model of the Ontology for Biomedical Investigations to represent strengths of active ingredients and then analyzed RxNorm to extract excipient and strength information and modeled them according to the results of our analysis. We also analyzed and defined dispositions of molecules used in aggregate as active ingredients to bind cytochrome P450 isoenzymes.

**Results:**

Our analysis of excipients led to 17 new classes representing the various roles that excipients can bear. We then extracted excipients from RxNorm and added them to DrOn for branded drugs. We found excipients for 5,743 branded drugs, covering ~27 % of the 21,191 branded drugs in DrOn.

Our analysis of active ingredients resulted in another new class, active ingredient role. We also extracted strengths for all types of tablets, capsules, and caplets, resulting in strengths for 5,782 drug forms, covering ~41 % of the 14,035 total drug forms and accounting for ~97 % of the 5,970 tablets, capsules, and caplets in DrOn.

We represented binding-as-substrate and binding-as-inhibitor dispositions to two cytochrome P450 (CYP) isoenzymes (CYP2C19 and CYP2D6) and linked these dispositions to 65 compounds. It is now possible to query DrOn automatically for all drug products that contain active ingredients whose molecular grains inhibit or are metabolized by a particular CYP isoenzyme.

DrOn is open source and is available at http://purl.obolibrary.org/obo/dron.owl.

## Background

In previous work, we built the Drug Ontology (DrOn) to support comparative effectiveness research use cases and reported on its theoretical basis, the methodology we used to build it, and its ability to meet the use cases [1–3]. Motivated by critiques and requests from end-users of DrOn of its representation of ingredients, we describe how we have improved the accuracy and coverage of our representation of ingredients.

The work involved three major components. The first component was the inclusion of excipients. Although active ingredients and their strengths have obvious effects on the efficacy of a drug, excipients also influence drug effects in significant ways [4–6]. Additionally, it is not uncommon for excipients to cause allergic reactions in patients [7, 8]. The second component was the improvement and extension of the representation of active ingredients, including the addition of strength information. The last component was representing for the first time in an open-access, machine-readable ontology the binding disposition of certain molecules to cytochrome P450 (CYP) isoenzymes as substrates and / or inhibitors.

## Methods

In Hogan et al. [1], we differentiated between excipients and active ingredients but did not define or represent their differences explicitly. To do so, we first conducted an ontological analysis of the roles various ingredients have in drug products. We also represented strengths of active ingredients according to the value specification model of the Ontology for Biomedical Investigations (OBI) [9]. We documented and reviewed our definitions and proposed classes and their axiomatizations on the DrOn wiki page [10]. Once complete, we then analyzed RxNorm [11] to extract excipient and strength information and modeled them according to the results of our analysis.

### Analysis of excipients and method of extracting them from RxNorm

We reviewed publicly available sources of information about the various roles of excipients and conducted an ontological analysis of them from the realist perspective. Excipients have numerous roles that aid in the manufacture, administration, identification, and preservation of drug products. To represent these roles, we defined the following and included them in DrOn: *excipient role, lubricant excipient role, glidant excipient role, anti-adherent excipient role, anti-friction excipient role, binding excipient role, coating excipient role, protective coating excipient role, enteric coating excipient role, administration coating excipient role, flavor coating excipient role, lubricant coating excipient role, color excipient role, flavor excipient role, disintegrant excipient role, preservative excipient role, sorbent excipient role,* and *vehicle excipient role.* We present the results of our ontological analysis, including textual and axiomatic definitions of these terms in the Results section.

RxNorm contains excipient information that it obtains from Structured Product Labels (SPLs). SPLs are a digital form of the physical product label that the Food and Drug Administration (FDA) collects from drug manufacturers. RxNorm includes information extracted from SPLs and stores it with a source abbreviation (used to identify the source of the information) of ‘MTHSPL’. RxNorm includes a ‘has_inactive_ingredient’ relationship extracted from the SPLs, which we used to identify the excipients for drug products in DrOn. Since DrOn previously only contained information from RxNorm under the source abbreviation ‘RXNORM’—which is data collected from the other sources and then normalized—we needed to match the MTHSPL atoms to the appropriate RxNorm concepts and then to the appropriate DrOn entities. It should be noted that the MTHSPL data is denoted source restriction level 0 in RxNorm, meaning it is licensed for creation of derivative open source works.

We also make extensive use of Semantic Clinical Drugs (SCDs) and Semantic Branded Drugs (SBDs) in RxNorm. Each SCD represents a unique combination of active ingredients, their strengths, and dose form. An SBD represents everything that an SCD represents plus information about a drug product’s trade name.^1^ Both SCDs and SBDs are the result of RxNorm’s normalization process, and thus are assigned concept identifiers (RxCUIs).

Using the April, 2015, release of RxNorm, we:

1. Found all the atoms in the RXNREL table that have a source abbreviation of ‘MTHSPL’ and a relationship type of ‘has_inactive_ingredient’.
2. Mapped both atoms to the appropriate RxNorm concept unique identifier (RxCUI).
3. Mapped the RxCUIs to atoms within the RXNCONSO table that have a source abbreviation of ‘RXNORM’ and a term type of ‘IN’ (for ingredients) or ‘SBD’ (for drugs).
4. Mapped the RxCUIs to DrOn drug product and ingredient classes that have the same RxCUI annotated on them.

This process gave us a mapping that connected branded drugs in DrOn to various excipient ingredients. Because we used unique identifiers from both DrOn and RxNorm (RxCUIs) to create this mapping, the process was straightforward, and required no manual resolution of ambiguity.

We excluded excipients linked to SCDs in RxNorm because we found that multiple generic and branded products extracted from SPLs were linked to SCDs but not SBDs, resulting in SCDs being linked to all the excipients of many drug products at the same time. For example, ‘dimethicone 10 MG/ML Topical Cream’ (RxCUI 200010) is associated with 39 different SPL drug products, including many branded drugs like ‘Proshield Glove Skin Protectant’ (RxAUI 4232431) or ‘Better Than Nature Eye Essence’ (RxAUI 4660113), for which there does not also exist in RxNorm a SBD. Future work involves representing these products distinctly in DrOn.

### Analysis of active ingredients and extracting their strengths from RxNorm

Although in Hogan et al. [1], we recognized the active ingredient as being a scattered molecular aggregate as defined and represented in the Ontology of Biomedical Investigations, the Web Ontology Language (OWL) representation of DrOn lagged behind this recognition. Our first major change, then, was to update the OWL representation of active ingredients from, for example:: (has_proper_part**some**ramipril) was updated to (has_proper_part**some**(‘scattered molecular aggregate’**and**(‘has granular part’**some**ramipril))).

The second update was to define ‘active ingredient’ as a role (see Results) and assert that the scattered molecular aggregate is the bearer of this role:

has_proper_part**some**(‘scattered molecular aggregate’**and**(‘has granular part’ some ramipril)**and**(‘is bearer of’**some**‘active ingredient role’))

The third update was to begin capturing strength information starting with the most prevalent and easiest case: tablets, capsules, and caplets. DrOn already contains all of the active ingredients found within RxNorm with a source abbreviation of ‘RXNORM’. In RxNorm, strengths are related to Semantic Drug Components (SCDCs), which are not represented in DrOn. RxNorm creates one SCDC per unique combination of active ingredient and strength and also relates a drug to its active ingredients via SCDCs with a consists_of relationship. We therefore carried out the following steps to map the active ingredients of drug products in DrOn to their appropriate strengths. We did this using the April, 2015, version of RxNorm as follows:

1. Mapped the clinical drugs within DrOn to RxNorm concepts in the RXNCONSO table with a source abbreviation of ‘RXNORM’ and a term type of ‘SCD’ using the annotated RxCUI.
2. Mapped the SCDs from the previous step to the appropriate concepts with a source abbreviation of ‘RXNORM’, a relationship of ‘consists_of’, and term type of ‘SCDC’ using the RXNCONSO and RXNREL tables.
3. Mapped the SCDC concepts from the previous step to the appropriate concepts with a source abbreviation of ‘RXNORM’, a relationship of ‘has_ingredient’, and term type of ‘IN’ using the RXNCONSO and RXNREL tables.
4. Mapped the IN concepts to the ingredients within DrOn using its RxCUI.
5. Pulled out the strength of the SCDC from the RXNSAT table using the ‘RXN_STRENGTH’ attribute name.

This process gave us a mapping between clinical drug, ingredient, and strength that we then used to build the OWL representation as illustrated below.

In DrOn, we place branded drug classes (corresponding to SBDs) as subclasses of classes that represent preparations of specific active ingredients, their strengths, and dose form (corresponding to SCDs). Thus, we only needed create axioms representing strengths at the SCD-equivalent level since these axioms are inherited by classes further down the hierarchy and thus apply to the branded drugs.

## Results

Our work has three key contributions: 1) a realist analysis and resulting ontological representation of drug excipients and the various roles they play, 2) a realist analysis of active ingredients and their strengths, and 3) a realist analysis of cytochrome P450 isoenzyme binding. In the rest of this section, we will describe them in detail.

### Realist analysis of drug excipients

The excipients used in drug products have varied roles. We define an **excipient role** as *a role of a scattered molecular aggregate in aiding the manufacture, prolonging the shelf life, aiding the identification, or ensuring proper administration of a drug product*.

Before creating a new term, we surveyed other OBO Foundry resources for existing terms that met our needs. The Chemical Entities of Biological Interest (ChEBI) ontology [12] defines an excipient role as *a generally pharmacologically inactive substance that is formulated with the active ingredient of a medication*.

This definition would seem to be inline with our usage, but the term seems to be used within ChEBI to apply to individual molecules rather than aggregates, meaning every molecule of magnesium stearate in some drug tablet has its own role to, for instance, decrease the adhesion between the other ingredient molecules and the manufacturing machinery. Although it is true that each molecule has some disposition that, in aggregate, leads to lower adhesion, a single molecule is not sufficient when added to a drug preparation by itself. Its intended usage, and thus its role, can only be realized in the aggregate, and thus we assign the role to the aggregate of all magnesium stearate molecules used in the manufacture of the drug product (not just those molecules remaining).

Furthermore, an excipient role as defined in ChEBI is too general. An excipient is added to a drug product with a specific intent, unless we are to count contaminants. If, in the process of manufacturing a drug product, some minor contaminant makes it into a gel capsule, it is not an excipient. Therefore, assigning the role of exicpient to all things formulated with the active ingredient is too broad.

**Fig. 1:**
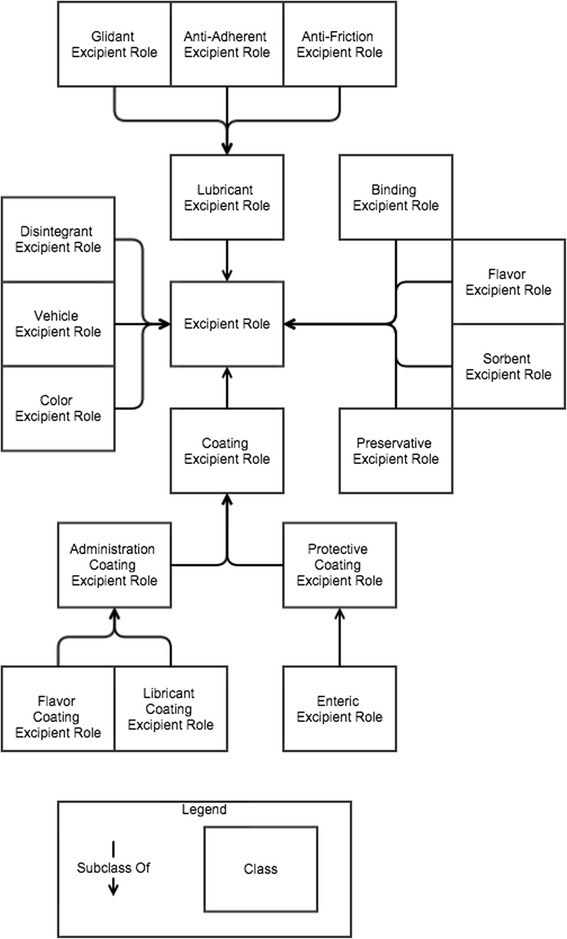
Excipients. The various excipient roles and their is-a relationships

In addition to a general excipient role, we have identified sixteen specific subtypes of excipients based on specific uses. Figure 1 shows the various types of excipient roles and the relations between them.

Lubricant excipient role: *An excipient role that is realized by a process of drug administration or a process of drug manufacturing and results in either 1) decreased adhesion between drug ingredients and manufacturing equipment or between drug ingredients and some part of an organism; 2) decreased friction between drug ingredients and manufacturing equipment or between drug ingredients and some part of an organism; or 3) decreased cohesion among particles within the drug preparation*.

Lubricant excipients are added to drug preparations to prevent ingredients from sticking to themselves (cohesion) and to other things with which they come into contact (adhesion). Common lubricants are minerals like magnesium stearate. There are three major subtypes of lubricants: glidants (glidant excipient role), anti-adherents (anti-adherent excipient role), and anti-friction lubricants (anti-friction excipient role). In defining the three subtypes, we make the distinction between adhesion (which is a steady or firm atachment) and friction (which is the force that provides resistance to relative motion). To see the difference consider a wet piece of paper: it will adhere to a plate of glass, but offer minimal friction to movement along the glass.

Glidant excipient role: *A lubricant excipient role that is realized by a process of drug administration or a process of drug manufacturing and results in decreased cohesion or friction among particles within a drug preparation*.

A glidant is added to a drug product to reduce cohesion and interparticle friction. Common glidants are talc and magnesium carbonate.

Anti-adherent excipient role: *A lubricant excipient role that is realized by a process of drug administration or a process of drug manufacturing and results in decreased adhesion between drug ingredients and manufacturing equipment or between drug ingredients and some part of an organism*.

Anti-adherents are added to drug products to decrease the tendency of drug molecules to adhere to manufacturing equipment or some body part such as the throat or esophagus during swallowing.

Anti-friction excipient role: *A lubricant excipient that is realized by a process of drug administration or a process of drug manufacturing and results in decreased friction between drug ingredients and manufacturing equipment or between drug ingredients and some part of an organism*.

Anti-friction excipients are added to decrease either internal friction (i.e., friction between ingredient particles) or friction between the drug ingredients or product and some other object, such as manufacturing equipment or some body part.

Binding excipient role: *An excipient role that is realized by a process of drug manufacturing and results in increased volume or cohesion of the drug product.*

Binding excipients are added to drug preparations to 1) bind active ingredients together, and 2) increase the volume of the preparation (which is especially important for formulations with otherwise small volumes). Common binding agents are saccharides (like sucrose) or synthetic compounds like polyethylene glycol.

Coating excipient role: *An excipient role borne by an aggregate of molecules on the surface of a solid drug product that is realized by a process of delaying interaction between entities outside the drug product and the other ingredients in the drug product.*

Coatings are extremely common excipients, added to protect the drug preparation from destruction or contamination, to ease administration by making it easier to consume, or to improve flavor. There are five major subtypes of coating excipient.

Protective coating excipient role: *A coating excipient role that is realized by delaying denaturation, disintegration, or some other method of destruction of a drug preparation including its active ingredients.*

A protective coating acts against destruction or contamination of a drug preparation by keeping the other drug ingredients, especially active ingredients, away from potentially reactive substances like oxygen, water, and various forms of electromagnetic radiation (e.g., light).

Enteric coating excipient role: *A protective coating excipient role that is realized by a process of delaying release of one or more active ingredients from the drug product until some targeted time or location, typically the small or large intestine, within an organism.*

An enteric coating also protects the drug preparation from destruction or contamination, but also is designed to disintegrate on a controlled timeline or in a particular place. For instance, some enteric coatings are designed to withstand the relatively high PH of the stomach, but break down in the relatively low PH of the large intestine, allowing an ingredient that would otherwise be destroyed by or absorbed by the stomach to be absorbed in the intestine.

Administration coating excipient role: *A coating excipient role that is realized by facilitating a process of drug administration*.

An administration coating is one that somehow improves administration of the drug, by for example making insertion or consumption of the drug easier or masking undesirable flavors.

Flavor coating excipient role: *An administration coating excipient role that is realized by a drug manufacturing process that results in the drug product bearing a particular flavor quality.*

Flavored coatings make it more palatable to consume a drug product by improving its taste, often by masking the unpleasant taste of the active ingredients.

Lubricant coating excipient role: *An administration coating excipient role that is realized by decreased friction between the drug preparation and some part of an organism during drug administration*.

A lubricant coating makes it easier to consume or insert a drug product by decreasing the friction or adhesion between the drug preparation and some body part such as the throat or esophagus.

Color excipient role: *An excipient role that is realized by a process of drug manufacturing that results in a particular, desired color quality of the drug product.*

Colored excipients are added to a drug preparation to make various kinds of drugs more easily identifiable by sight to decrease the possibility of using the wrong dosage or wrong drug product altogether.

Flavor excipient role: *An excipient role that is realized by a process of drug manufacturing that results in the drug product bearing a particular flavor quality.*

Like a flavored coating, a flavored excipient is added to the drug preparation to make it more palatable. This is especially important for drug products targeted towards children to make administration easier.

Disintegrant excipient role: *An excipient role that is realized by a process of drug administration followed by the drug product breaking apart*.

A disintegrant is added to a drug preparation to cause it to break apart whenever it is introduced to moisture. A disintegrant can improve administration (such as oral medications that dissolve in the mouth) or improve uptake of active ingredients for example, in the intestine.

Preservative excipient role: *An excipient role that is realized by increasing the duration of time that a drug product is effective or by inhibiting contamination of the drug product with a microorganism*.

Preservatives are added to a drug preparation to increase the lifetime of the drug preparation. Examples include antioxidants such as ascorbic acid that prevent oxidation-reduction reactions that change active ingredients into inactive compounds and methyl paraben which is an antimicrobial preservative.

Sorbent excipient role: *An excipient role that is realized by its bearer binding with water in the environment to prevent water binding with other ingredients in the drug product.*

Sorbents are added to protect the drug preparation from destruction or disintegration by water. A common example is a desiccant, which is a sorbent that prevents absorption of water into the drug product.

Vehicle excipient role: *An excipient role that is realized by a completed process of the active ingredient reaching its intended destination during drug administration*.

Generally, vehicles are the media in which the active ingredient is dispersed to facilitate the active ingredient reaching its intended target tissue. For example, active ingredients that exist in solid form such as a powder cannot be directly injected intravenously without causing damage to veins or becoming emboli that cause damage to the lungs. Thus they are dissolved in solution for safe and proper administration. Other examples of vehicles include creams, ointments, lotions, gels, and solvents for ophthalmic, otic, and oral solutions.

Having reviewed and defined the major subtypes of excipients, we next illustrate how we represent molecular aggregates and their excipient roles in the DrOn OWL files. Consider a drug tablet that contains povidone and pregelatinized starch as excipients. We axiomatize this tablet as follows:

tablet**and**(has_proper_part**some**('scattered molecular aggregate'**and**

(has_granular_part**some**povidone)**and**

(bearer_of**some**'binding excipient role')))**and**

(has_proper_part**some**('portion of pregelatinized starch'**and**

(bearer_of**some**'binding excipient role')))

Our extraction of excipient information from RxNorm resulted in the representation of excipients for 5,743 branded drugs, covering ~27 % of the 21,191 total number found in DrOn. There are a total of 35,455 different drug product–excipient relationships. By comparison, there are 22,845 relationships between drug products and active ingredients. The main reasons there are fewer relationships between drugs and active ingredients than there are between drugs and excipients is that there are fewer active ingredients and that active ingredients are specified at a higher level in the taxonomy of drugs. Active ingredients are defined at the level of clinical drug form (for example, furosemide oral tablet) whereas excipients exist at the level of branded drug (more specific than clinical drug form), because each brand of a drug product such as furosemide 20 mg oral tablet typically contains a different set of excipients.

### Realist analysis of active ingredients

Although DrOn has always included active ingredients, we have updated the representation to more accurately reflect reality and to allow us to add strengths to drug products. To do so, it was necessary to represent **active ingredient role**, which we define as *a role borne by an aggregate of molecules that is a proper part of a drug product and that is realized by (1) administration of the drug to an organism followed by (2) some change in the structure or functioning of some part of the organism or its endosymbiotic organisms.*

This definition meets several criteria we identified during our analysis of active ingredients. First, it is a realizable entity. Note that an active ingredient does nothing until and unless the drug product is appropriately administered. Second, it is a role rather than a disposition (or, more specifically, a function). Some ingredients can serve as either an excipient or an active ingredient depending on the specific drug product. For example, calcium carbonate is an active ingredient in certain antacid tablets, but an excipient in other products. Furthermore, calcium carbonate neither evolved nor was designed to neutralize acids (a key criterion of functions per BFO). Of course, there is some disposition at the molecular level that the realization of the active ingredient role depends on; in the case of calcium carbonate, its physical makeup causes it to react with strong acids, releasing carbon dioxide. But this disposition inheres in each individual molecule of calcium carbonate whereas the active ingredient role inheres in the entire aggregate in the tablet: clinically signficant acid neutralization occurs only with the aggregate delivered via the tablet.

We represent the *active ingredient role* in OWL in a manner similar to how we represent the excipient role. We represent a drug tablet that has acetaminophen as an active ingredient with a strength of 325 MG as the following:

tablet**and**(has_proper_part**some**('scattered molecular aggregate'**and**

(has_granular part**some**acetaminphen)**and**

(bearer_of**some**‘active ingredient role')**and**

(bearer_of**some**(mass**and**

(has_specified_value ‘325’)**and**

(has_measurement_unit**value**milligram)))))

We added strengths to 5,782 clinical drugs, covering ~41 % of the 14,035 total number, and accounting for ~97 % of the 5,970 tablets, capsules, and caplets in DrOn. Representing strengths for drug products in other dose forms (e.g., injectable solutions, creams, lotions, etc.) is future work.

### Realist analysis of cytochrome P450 isoenzyme binding

When a particular molecule binds to one of the isoenzymes in the cytochrome P450 (CYP) family, it does so as substrate, inhibitor, or both. Induction of CYP isoenzymes does not involve binding to individual enzyme molecules themselves, but rather it involves increasing transcription of CYP isoenzyme genes so that more individual enzymes come into existence. We could not find in the literature any case where molecular binding of a small molecule to a CYP isoenzyme induced or facilitated the activity of the isoenzyme.

We therefore represent binding of a small molecule to a CYP isoenzyme as a disposition of the molecule. It is not a function because the small molecule neither evolved nor was designed to have this binding effect. It is not a role because the tendency to bind is internal to the physical structure of the molecule itself. Nothing external or socially-designated causes the binding tendency to exist (note: we are loosly using the word ‘tendency’ here to equate to what BFO calls ‘realizable entity’).

We subdivide the binding disposition based on whether its realization results in transformation of the molecule into another type of molecule (that is, metabolism of the molecule into something else) versus whether its realization causes inhibition of the isoenzyme in metabolizing other small molecules. We note that many types of molecules used as active ingredient drugs have both substrate and inhibitory dispositions (for example, esomeprazine is both an inhibitor and substrate of CYP2C19).

To represent enzyme binding, we identified an *enzyme binding* class in the Gene Ontology (GO) and imported it into DrOn via the Minimum Information to Reference an External Ontology Term (MIREOT) methodology [13]. Its definition is the following: *interacting selectively and non-covalently with any enzyme.* For completeness, we also import *protein binding* and *binding,* the parent and grandparent of *enzyme binding*, respectively, into DrOn.

For binding to the active site of an enzyme, we were unable to identify a candidate term from any other realist ontology. Thus, we created the term *enzyme active site binding disposition*, which we defined as *a disposition borne by some molecular entity that is realized by binding to some enzyme and being destroyed in a process that realizes some function of said enzyme*. Similarly, we could not find and therefore created the term *function-inhibiting enzyme-binding disposition*, and defined it as *a disposition borne by some molecular entity that is realized by 1) binding to some enzyme and 2) subsequent inability of the active site of the enzyme to bind its substrate(s).*

We represented substrate and inhibitory binding dispositions for CYP2C19 and CYP2D6, because these are the major two isoenzymes targeted by the personalized medicine program at the University of Florida [14, 15]. We represented these in OWL by adding axioms as follows, using CYP2C19 inhibitory disposition as an example, to the molecular entities for which they are applicable:

subclassOf (bearer_of**some**function-inhibiting CYP2C19 binding disposition)

In total, we added CYP2C19 and CYP2D6 binding dispositions to 65 molecules, with some of them being the bearer of both an inhibitory and substrate definition (Table 1). Our source of data for the types of molecules that bear the particular types of binding dispositions was the P450 drug interaction table of the Indiana University School of Medicine [16].

**Table 1:** Number of CYP binding dispositions of various types in DrOn. The total number of molecular entities is 65; many have more than one disposition

	Substrate	Inhibitory
CYP2C19	26	16
CYP2D6	45	36

## Discussion

We have significantly updated and improved the representation of ingredients in the Drug Ontology. In the process, we have defined a number of key terms in DrOn including ‘active ingredient role’, ‘excipient role’, terms for numerous subtypes of excipient, and various terms for cytochrome P450 substrate and inhibitory binding. This representation enables automated algorithms to distinguish active ingredients from excipients in drug products, as well as determine the strength of drug products that are capsules, tablets, and caplets. Given that excipients have important clinical consequences, including hypersensitivity reactions, their inclusion could help improve research on drug products, pharmacogenomics, and clinical decision support.

A key use of DrOn is in the improvement and standardization of knowledge of drug-drug interactions (DDIs) [17]. This work requires accurate representations of active ingredients with strengths and excipients since they impact the potential for, likelihood, and severity of interactions. Our work on CYP isoenzymes further enables query of DrOn for all drug products that contain an ingredient whose molecular grains bind particular CYP isoenzymes. A researcher could use these representations, for example, to identify all patients who are taking one drug that inhibits a given isoenzyme and another drug that is metabolized by it, and thus is at risk for adverse effects of the latter drug (that is, the inhibition caused by the first drug will reduce the metabollism of the second drug, leading to increased levels and thus increased risk of toxicity).

Other work seeks to infer DDIs based on common properties including the structure of compounds [18]. Specifically, if some but not all compounds with a given property or structure *X* are asserted to have a DDI with some compound *Y*, then this method identifies the remaining compounds with *X* as candidates for also having a DDI with *Y*. As DrOn is increasingly used for DDI representation, it will be interesting future work to compare this method applied to DrOn-based DDI representations vs. other artifacts.

For DrOn’s representation of strengths, we were able to reuse the value specification of the Ontology of Biomedical Investigations as well as its object and datatype properties. We used the MIREOT Protégé plugin we developed [13] to include these properties as well as the units of measure required.

While adding excipients, we discovered that there was a significant sparsity of branded drugs in RxNorm with excipient information. The reason is likely that RxNorm began incorporating SPLs, the current source of excipient information, only recently in 2012. Additionally, RxNorm has mapped many drugs with FDA SPLs to Semantic Clinical Drugs only. For example, ‘*dimethicone 10 MG/ML Topical Cream*’ (RxCUI 200010) is associated with 39 different SPL drug products, including many branded drugs like ‘*Proshield Glove Skin Protectant*’ (RxAUI 4232431) or ‘*Better Than Nature Eye Essence*’ (RxAUI 4660113). This SCD has around 170 different excipients associated with it. Another example is ‘*Dextromethorphan Hydrobromide 2 MG/ML / Guaifenesin 20 MG/ML Oral Suspension*’ (RxCUI 1605844), which is associated with ‘*Tussin Cough and Chest Congestion DM Adult*’ (RxAUI 6836489). The excipients linked to these Semantic Clinical Drugs appear to be a superset of all the excipients of the SPL-derived drug products that RxNorm links to the SCD. Because RxNorm does not represent generic drug products, the excipients of all generic products also appear to be linked to the SCD. Of course, these observations are likely related. Further analysis is required.

In the process of defining the active ingredient role, we added the capability to represent pharmaceutical strength. We began with tablets, capsules, and caplets because they represent the total quantity of active ingredient, which is simpler to represent than concentrations. For other dose forms, RxNorm specifies the quantity of active ingredient per unit of drug product (e.g., per milliliter of solution, per gram of ointment) and the total quantity of drug product (e.g., 5 mL vial, 25 g tube of ointment) is not always available from which the total mass of active ingredients could be derived.

### Future Work

We have three primary directions for future work. First, we intend to increase coverage of excipients and strengths of active ingredients. Our strength coverage for the dose forms we used in this analysis is sufficiently high, but we still need to work out the representation and then extract strength information for other dose forms, which are expressed as relative vs. total quantity of active ingredient. Additionally, we intend to tease out the excipients that are currently mapped to SCDs in RxNorm, which requires further analysis.

Second, we intend to represent the induction of CYP isoenzymes by particular active ingredients in drug products. The inductive effect is indirect through an increased rate of genetic transcription that creates additional copies of CYP isoenzymes, rather than through mere binding to the isoenzyme. It is therefore somewhat more complex to represent. It is also likely an aggregate effect as opposed a property of any individual molecule (although dispositions of the molecules are certainly involved along the way).

Third, we intend to represent therapeutic indications of drug products. We currently posit that a therapeutic indication is a function borne by a drug product that is realized by a process of administration to an organism, distribution of one or more active ingredients to some target tissue, and resulting in some physical change in the targeted tissue. However, this work requires further development of use cases and ontological analysis.

## Conclusions

In this paper, we describe three primary lines of work: 1) an update to our representation of active ingredients, including adding strengths; 2) a new representation of excipients; and 3) a new represention of substrate and inhibitory binding dispositions for CYP2C19 and CYP2D6. We created new terms and definitions for *excipient role* and sixteen different subtypes, the *active ingredient role*, and various terms to represent substrate and inhibitory binding dispositions for CYP2C19 and CYP2D6. We also reported on how these terms were used in the Drug Ontology, and made the updated representations available at http://purl.obolibrary.org/obo/dron.owl.

## Footnotes

1 And hence only branded drug products, and not generic drug products, of manufacturers are assigned RxCUIs.
